# HIV-1 Transmitting Couples Have Similar Viral Load Set-Points in Rakai, Uganda

**DOI:** 10.1371/journal.ppat.1000876

**Published:** 2010-05-06

**Authors:** T. Déirdre Hollingsworth, Oliver Laeyendecker, George Shirreff, Christl A. Donnelly, David Serwadda, Maria J. Wawer, Noah Kiwanuka, Fred Nalugoda, Aleisha Collinson-Streng, Victor Ssempijja, William P. Hanage, Thomas C. Quinn, Ronald H. Gray, Christophe Fraser

**Affiliations:** 1 MRC Centre for Outbreak Analysis and Modelling, Department of Infectious Disease Epidemiology, Imperial College London, London, United Kingdom; 2 School of Medicine, Johns Hopkins University, Baltimore, Maryland, United States of America; 3 National Institute of Allergy and Infectious Diseases, National Institutes of Health, Baltimore, Maryland, United States of America; 4 School of Public Health, Makerere University, Kampala, Uganda; 5 Rakai Health Science Program, Entebbe, Uganda; 6 Bloomberg School of Public Health, Johns Hopkins University, Baltimore, Maryland, United States of America; 7 Department of Infectious Disease Epidemiology, Imperial College London, London, United Kingdom; The Pennsylvania State University, United States of America

## Abstract

It has been hypothesized that HIV-1 viral load set-point is a surrogate measure of HIV-1 viral virulence, and that it may be subject to natural selection in the human host population. A key test of this hypothesis is whether viral load set-points are correlated between transmitting individuals and those acquiring infection. We retrospectively identified 112 heterosexual HIV-discordant couples enrolled in a cohort in Rakai, Uganda, in which HIV transmission was suspected and viral load set-point was established. In addition, sequence data was available to establish transmission by genetic linkage for 57 of these couples. Sex, age, viral subtype, index partner, and self-reported genital ulcer disease status (GUD) were known. Using ANOVA, we estimated the proportion of variance in viral load set-points which was explained by the similarity within couples (the ‘couple effect’). Individuals with suspected intra-couple transmission (97 couples) had similar viral load set-points (*p* = 0.054 single factor model, *p* = 0.0057 adjusted) and the couple effect explained 16% of variance in viral loads (23% adjusted). The analysis was repeated for a subset of 29 couples with strong genetic support for transmission. The couple effect was the major determinant of viral load set-point (*p* = 0.067 single factor, and *p* = 0.036 adjusted) and the size of the effect was 27% (37% adjusted). Individuals within epidemiologically linked couples with genetic support for transmission had similar viral load set-points. The most parsimonious explanation is that this is due to shared characteristics of the transmitted virus, a finding which sheds light on both the role of viral factors in HIV-1 pathogenesis and on the evolution of the virus.

## Author Summary

During the long period of asymptomatic infection with HIV-1 there is considerable variability in viral load set-point between infected individuals. Higher viral load set-points increase infectivity and decrease survival. Previous work has shown that the most commonly observed viral load set-points are those intermediate viral load set-points which lead to the largest number of opportunities to transmit HIV-1 in an infectious person's lifetime, balancing survival and infectiousness. This coincidence between the most common viral load set-points and the optimum for lifetime transmission could be the result of population-level selection acting on HIV-1. However, this could only have happened if viral load set-point is a heritable characteristic of the virus, i.e. if viral load set-points are similar between both partners of transmitting couples. By studying viral load set-points amongst heterosexual couples, we show that viral load set-points are similar in these couples. When we study only those couples with strong genetic support for transmission, their viral loads are even more similar than when we study the whole group. These results suggest that there are viral factors which are passed from one infected individual to the next which play a role in determining viral load set-point and that population-level selection could act upon these viral factors.

## Introduction

The severity of HIV-1 infection is thought to result from an interplay of factors in the host, the virus, and the environment (for instance the presence of co-infections). Much work has focused on resolving host genetic factors which contribute to virulence [1], while the possible role of viral genetic factors inherited with the virus and transmitted with infection remains largely unresolved. Differences between viral subtypes are uncontroversial: for example subtype D appears to be associated with faster rates of disease progression [2]–[8]. But whether or not differences exist between more closely related strains, within subtypes, has not been established.

The existence of heritable viral factors influencing disease progression, and their contribution relative to other factors, is of interest for at least two reasons. Firstly, such factors, if they exist, have implications for how the virus influences the course of infection within an infected person. Secondly, if viral factors exist which affect virulence and can be preserved from one infection to the next, then these factors will be subject to natural selection at the population level [9]. In this study we test for the existence of such factors by examining viral load set-points among transmitting couples. HIV-1 viral load set-point is a quantitative measure of viral RNA copies in peripheral blood during asymptomatic infection. Viral load set-point is commonly used as a surrogate measure of the virulence of an infection since it is negatively associated with the time to AIDS and death [10].

At present, limited evidence suggests that viral load set-point is regulated by viral factors, although to an extent this reflects a paucity of research on the topic. The existence of a specific recombinant form associated with high viral loads strongly suggests that viral factors can play a role in at least some circumstances [11], as does the demonstration of stable differences between SIV strains in the outcome of experimental infection of macaques [12]. Other experimental evidence includes differences between closely related HIV-1 strains in competition experiments [13], [14]. In cases of natural infection, an early study demonstrated a correlation between the time to AIDS among infected blood donor index cases and the recipients of their blood products [15]. The importance of unravelling the role of host and viral factors is illustrated by the strong correlation in viral loads which has been observed in mother-to child infections [16], which could be attributed to a combination of host and viral factors.

Finally, and most convincingly, a study of 115 HIV transmitting heterosexual couples in Zambia showed that 19% of variance in viral loads could be explained by shared homologous virus between couples (*p* = 0.03) [17]. This study suggested a role for viral factors in determining viral load, but has not been repeated.

It has been hypothesised that the observed distribution of viral load set-points could be the result of natural selection acting on viral factors in order to maximise opportunities for transmission [9]. This hypothesis arose from an epidemiological analysis of the quantitative dependence between viral load, infectiousness and the duration of asymptomatic infection. This study demonstrated that people with the most common viral load set-points are predicted to be the most productive in terms of onward transmission over the course of infection; lower viral loads are associated with a longer life expectancy and thus more opportunities for transmission, but this is offset by reduced infectiousness. Conversely, those with higher viral load set-points are more infectious, but progress to AIDS too quickly to produce as many onward infections over the whole course of their asymptomatic period. In other terms, the observed distribution of viral load set-point is consistent with an evolutionary life-history trade-off for the virus [9]. If this interpretation is correct, then the observed distribution of viral loads set-points and, by extension, virulence, could be the product of viral adaptation acting to maximise opportunities for transmission.

For this hypothesis to be correct, viral load set-point must be a heritable property, partly determined by the virus and preserved from one infection to the next. If it is not heritable, there is no way natural selection can act upon it. In this study we estimate heritability in viral load set-point within transmitting couples, and account for a number of important confounding factors. We estimate heritability as the proportion of variance in viral load set-point which is determined by infection with genetically similar virus for HIV-1-infected heterosexual couples identified in the Rakai District of south-western Uganda.

## Methods

### Study population

The study population was enrolled in the Rakai Community Cohort Study in the rural Rakai District of south-western Uganda. Study methods have been described in detail elsewhere [18], [19], but are briefly outlined here.

More than 12,000 consenting subjects aged 15–49 were interviewed in surveys conducted at 10–12 month intervals from 1994–2003. Participants provided written, informed consent; and were provided with condoms and voluntary HIV counselling and testing free of charge. Participants agreed to provide identifying information for their married or consensual partners which allowed retrospective linkage of couples. The study was approved by review boards at the Uganda Virus Research Institute, the AIDS Research Subcommittee of the Ugandan National Council for Science and Technology, Columbia University, and Johns Hopkins University. HIV prevalence in the cohort was 16.5%, and average annual HIV incidence was 1.5 cases/100 person-years [19].

Retrospective analyses identified 200 self-reporting sexual partners for whom there was evidence of seroconversion for one or both partners during the course of the study. The partner who was seropositive first was identified as the index case, and the other partner as the secondary transmission case. For some couples the ordering of events could not be identified because they both seroconverted within the same round of the study. For some of these concurrently infected couples, the partner reporting an external sexual relationship could be inferred to be the index individual.

Serum samples from venous blood provided at survey visits were tested for HIV-1 RNA levels quantified by a reverse-transcriptase polymerase chain reaction (RT-PCR) assay (Amplicor HIV-1 Monitor 1.5 assay, Roche Molecular Systems) with a lower detection limit of 400 copies/mL (2.6 log_10_ copies/mL). Antiretroviral therapy (ART) was not available in Rakai at the time of the study, but participants were offered free general health care and treatment for opportunistic infections.

During early infection and prior to AIDS and death, viral loads are elevated above the set-point. To exclude data from early infection, viral loads measured at the first visit with a positive serology following a previous visit with negative serology were excluded. To ensure measurements made during late infection were also excluded, viral loads from the last observation prior to death (up to a maximum of 12 months prior to death) were also excluded. Following these exclusions 112 couples were identified for whom suitable viral load measurements were available. For those individuals with more than one viral load measurement, the set-point was defined as the mean log_10_ viral load over eligible visits. Age at the time of measurement was also averaged.

HIV-1 subtype was determined for 171 individuals using a Multi-region Hybridization Assay (MHA_acd_) on serum samples [20] as previously described for this cohort [6], [21]. Samples were classified as subtype A, D, C and A/D recombinants. 8 couples whose subtypes were discordant (where subtypes were available for both individuals) were excluded from the statistical analysis.

For a subset of the couples viral sequence data from the gag (*p24*) and *gp41* regions were available for comparison in both partners to help identify transmitting couples. To decrease the risk of spurious linkages between sequences, *p24* and *gp41* sequences for the couples were analysed together with sequences from 511 other infected individuals in the cohort [22]; in total 620 *p24* and 614 *gp41* sequences were analysed (See Text S1 for Genbank accession numbers). For 603 of these, sequences at both loci were available for a particular individual. In these cases a phylogenetic analysis was conducted on the concatenate of the two sequences. A European subtype B virus, accession number EU786678.1, was used as the outgroup for all loci. The sequences are approximately 400 base pairs long, which is sufficient to cluster sequences for our purposes. Phylogenies were derived by maximum likelihood methods using a genetic substitution model chosen among many to best represent the data. The most appropriate substitution model was selected by comparing the rapid maximum likelihood fits in jModelTest v.0.1.1 [23], [24] by Akaike Information Criteria (AIC). The model selected was a general time reversible (GTR) nucleotide substitution model with a gamma distribution of rates (+G) and a proportion of invariant sites (+I) was used. The GTR+G+I model was the most suitable model among 88 candidate models for the concatenated sequences and *gp41* sequences by the Akaike Information Criterion (AIC). It was the third most appropriate model for *p24* (with ΔAICc = 12.2) but was also used for this locus for comparability between loci.

The phylogenetic analysis was conducted using RAxML 7.0.3 [25] which produced a maximum likelihood tree using a rapid bootstrapping algorithm (100 replicates) [26]. The bootstrap values written on to this tree were determined by a further 1000 bootstrap replicates produced by a rapid hill-climbing algorithm [27].

Concatenates of both loci were used to assess phylogenetic support for epidemiological linkage where they were available for both individuals (36 couples). For 14 couples, sequence data were only available for both individuals at *p24*, and for 8 couples data were only available for both individuals from *gp41*. In these cases it was only possible to perform the analysis based on single loci.

Couples were considered to be strongly linked if their sequences were monophyletic and the clade had bootstrap support of greater than or equal to 80%. This condition was imposed on both single locus and concatenated sequences to account for the possible effects of recombination in distorting the phylogenetic signal. Our approach to determining linkage within couples is thus conservative. Couples were considered to have no support for linkage if their sequences were polyphyletic.

To analyse the data on viral load set-points within transmitting couples, we performed our analysis on two groups. The first group included all couples, with and without genetic data, but excluding both those with sequence data who had no genetic support for linkage and those with discordant subtypes determined by MHA_acd_. The second subgroup included couples with strong genetic support for linkage (monophyletic with greater than 80% bootstrap support).

Symptoms of genital ulcer disease (GUD) over the interval prior to sample collection were ascertained via interview, and by physical examination for ulcers reported to be present at the time of a study visit. GUD has previously been found to be a significant predictor of viral load in this cohort [28]. If either or both partners had GUD which raised their viral loads during the study period this might confound the correlation of viral loads between individuals within couples. A report of any GUD in the six months prior to or at the time of viral load measurements was considered to be presence of GUD.

Since the data used in this study were not collected with the analysis presented here in mind, there are incomplete data on sequencing and epidemiological data. This may lead to unidentified biases in the data.

### Statistical analysis

We used analysis of variance (ANOVA) to test whether there was greater similarity in viral load set-points of individuals within transmitting couples than between all individuals. In other words, we decomposed the variance in viral load set-point into the sum of within-couple variance and between-couple variance. To perform ANOVA, a general linear model was formulated with a regression coefficient for each couple (see Text S2). In a first unadjusted analysis, the best estimate of these regression coefficients is the mean of the viral load set-points of two individuals in a couple. The significance test is then a comparison of this model (where each viral load is predicted by the coefficient for the couple) versus the null model (where there is only one coefficient, the overall mean for all individuals).

The strength of the effect is measured by *R* ^2^, the proportion of variance explained by the model. Since one parameter is introduced for each couple, a proportion of variance is explained spuriously due to decreased residual degrees of freedom. The adjusted *R* ^2^, denoted 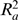, is defined as the proportion of the remaining variance explained and accounts for this spurious effect.

To adjust for possible confounders, the general linear model was extended to include the effects of gender, age and GUD status, which have all been previously shown to affect viral load set-point within this study population [28], and role in transmission (index or secondary case).

Since the biological origin of any similarity in viral loads is hypothesized to be due to similarity in viral genotypes between transmitting individuals, the measured association may be interpreted as an estimate of the effect of viral genotype on viral load set-point. In this context, the study design is analogous to pedigree studies in classical genetics which are used to study the association between genotype and phenotype [29]. Broad-sense heritability is defined as the ratio of genotypic variance to phenotypic variance. In our study, heritability is estimated by the ratio of variance in viral load set-points within transmitting couples, to variance in viral load set-points in the population as a whole [29]. In other words, heritability and *R^2^* are equivalent concepts.

We thus estimate heritability, as 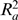 for the single factor model. The estimate can further be adjusted for confounders, denoted here by 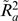 (see Text S2 and [30]).

The validity of our statistical approach is supported by the observation that the *p*-values obtained from the unadjusted analysis were equal to the proportion of permutation tests (repeatedly sampling and re-linking individuals into random pseudo-couples) which gave the same or larger 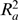, and also to *p*-values obtained by comparing the distribution of differences in viral loads within and between couples, thus confirming the validity of ANOVA to analyse these data (analysis not shown).

A related question of interest is the extent to which the viral load set-point of one individual can be used to predict the set-point of the person they infect. The strength of association in a unidirectional analysis (the correlation coefficient, 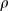) is equal to heritability, a relation which can be shown to hold exactly for viral loads distributed according to a bivariate Normal distribution, and also holds for the data analysed here (not shown) [29].

As outlined above, we performed this analysis on the large group of couples with moderate support for transmission and a subset with strong genetic support for transmission. The first group included all couples for whom there was epidemiological linkage and, where data was available, at least moderate genetic support for transmission. The second, more conservative, subgroup included only those with strong genetic support for transmission. We were thus able to investigate whether the signal became stronger when stricter inclusion criteria were imposed.

## Results

### Phylogenetic analysis

**Figure 1:**
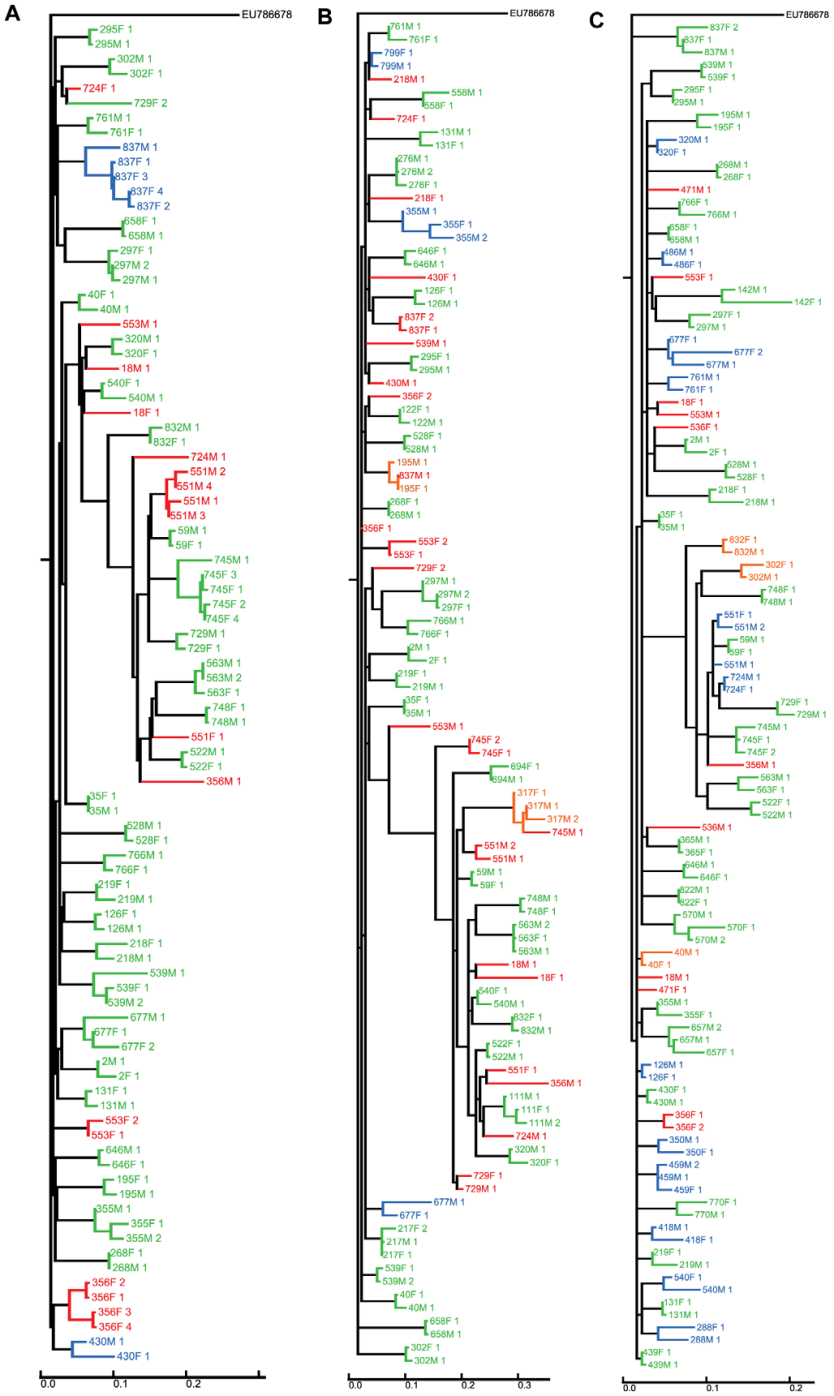
Clustering of sequences from the couples for whom sequence data was available. Sequences from the couples were analysed together with sequences from 511 other infected individuals in the cohort (‘filler sequences’) to prevent spurious linkage due to independent infections with similar circulating virus. For the sake of clarity, filler sequences are not shown in this figure (the full trees are shown in Figure S1, S2 and S3). Sequences from couples are categorised as polyphyletic (red), monophyletic with bootstrap <80% (blue) or monophyletic with bootstrap ≥80% (green). Additional couples who are monophyletic but for one invading sequence are indicated in orange. Black indicates a sequence from a couple which are monophyletic for sequences taken at another timepoint. **A** Concatenated sequences, **B** *gp41* only **C** *p24* only.

The phylogenetic trees used to identify the level of linkage between couples are shown in Figure 1. The additional sequences included to prevent spurious linkage have been excluded from the figure for clarity (full trees are shown in Figure S1, S2 and S3). The outcome of the phylogenetic clustering analysis and resulting inclusion criteria for the ANOVA are summarised in a flow chart (Figure S4).

Of the 35 couples with data available for both individuals at both *p24* and *gp41*, 31 were monophyletic, with 29 showing greater than 80% bootstrap support. The remaining 4 couples were polyphyletic. Of the 29 couples strongly linked on the concatenated tree, 16 showed strong support for linkage at both loci in the single locus trees.

14 couples had sequence data available for both partners at *p24* alone. Of these, 12 were monophyletic, 7 of which had greater than 80% bootstrap support, and 2 were polyphyletic (Figure 1B). Of the 8 couples with data available for both partners at *gp41* alone, 7 were monophyletic, with 6 showing greater than 80% bootstrap support (Figure 1C).

Overall, 29 of the 57 couples with viral sequence data showed strong support for intra-couple transmission based on genetic linkage (monophyletic with >80% bootstrap support on a single locus or multiple loci where available). There were indications that 8 couples (14%) did not transmit to each other and the remaining 21 couples were indeterminate (37%). The couples with strong support for transmission have distinctly closer tree distances than the rest of the sample (Figure S5).

Following the phylogenetic and subtype analysis, the statistical analyses were performed on 97 couples with moderate support for transmission (Figure S4) and a subgroup of 29 couples who had strong support for transmission.

### Viral loads

The average log_10_ RNA viral load set-point was 4.39 log_10_ cps/mL with values in the range 2.60 log_10_ cps/mL (the limit of detection) to 7.14 log_10_ cps/mL (Table 1). The average duration of follow up was just under a year (352 days) and 3 viral load datapoints from which to calculate set-point. Nearly half of individuals had only one valid viral load datapoint (86 of 194, 44%). The majority of couples were infected with subtype D viruses (63% of all individuals), with subtype A the second most common subtype (12%) (Table 1). Amongst individuals for whom GUD status was known, the majority were GUD negative, across all groups, and no significant association was found between GUD within couples (*p* = 0.14).

**Table 1:** Mean log_10_ HIV load set-point.

	Couples with moderate support for transmission (97 couples)		Subgroup of couples with strong support for transmission (29 couples)	
Variable	No. (%)	HIV load, mean log10 copies/mL (SD)	No. (%)	HIV load, mean log10 copies/mL (SD)
*All*	194 (100%)	4.39 (0.84)	58 (100%)	4.51 (0.79)
*Gender*				
Male	97 (50%)	4.42 (0.85)	29 (50%)	4.46 (0.85)
Female	97 (50%)	4.36 (0.84)	29 (50%)	4.57 (0.73)
*Age, years*				
15–24	40 (21%)	4.45 (0.74)	14 (24%)	4.46 (0.57)
25–29	52 (27%)	4.40 (0.77)	20 (34%)	4.48 (0.86)
30–39	53 (27%)	4.59 (0.70)	18 (31%)	4.78 (0.71)
40–64	26 (13%)	4.63 (0.80)	4 (7%)	4.21 (1.03)
Missing	23 (12%)	3.52 (1.02)	2 (3%)	3.42 (1.16)
*GUD*				
Present	28 (14%)	4.52 (0.73)	14 (24%)	4.69 (0.76)
Absent	118 (61%)	4.57 (0.78)	39 (67%)	4.55 (0.75)
Missing	48 (25%)	3.87 (0.87)	5 (9%)	3.74 (0.85)
*Subtype*				
A	24 (12%)	4.52 (0.67)	10 (17%)	4.50 (0.71)
C	1 (1%)	4.70		
D	122 (63%)	4.53 (0.76)	36 (62%)	4.64 (0.75)
Recombinant	28 (14%)	4.37 (0.81)	12 (21%)	4.13 (0.89)
Missing	19 (10%)	3.36 (0.95)		
*Transmission*				
Index partner	75 (39%)	4.52 (0.72)	23 (40%)	4.61 (0.63)
Secondary case	75 (39%)	4.35 (0.87)	23 (40%)	4.60 (0.85)
Missing	44 (23%)	4.24 (0.97)	12 (21%)	4.15 (0.91)

Viral load set-point for individuals in the 97 seroconverting couples with moderate support for transmission and in the subgroup of 29 couples with strong genetic support for transmission by sex, age, genital ulcer disease (GUD) status, subtype and role in transmission.

**Figure 2:**
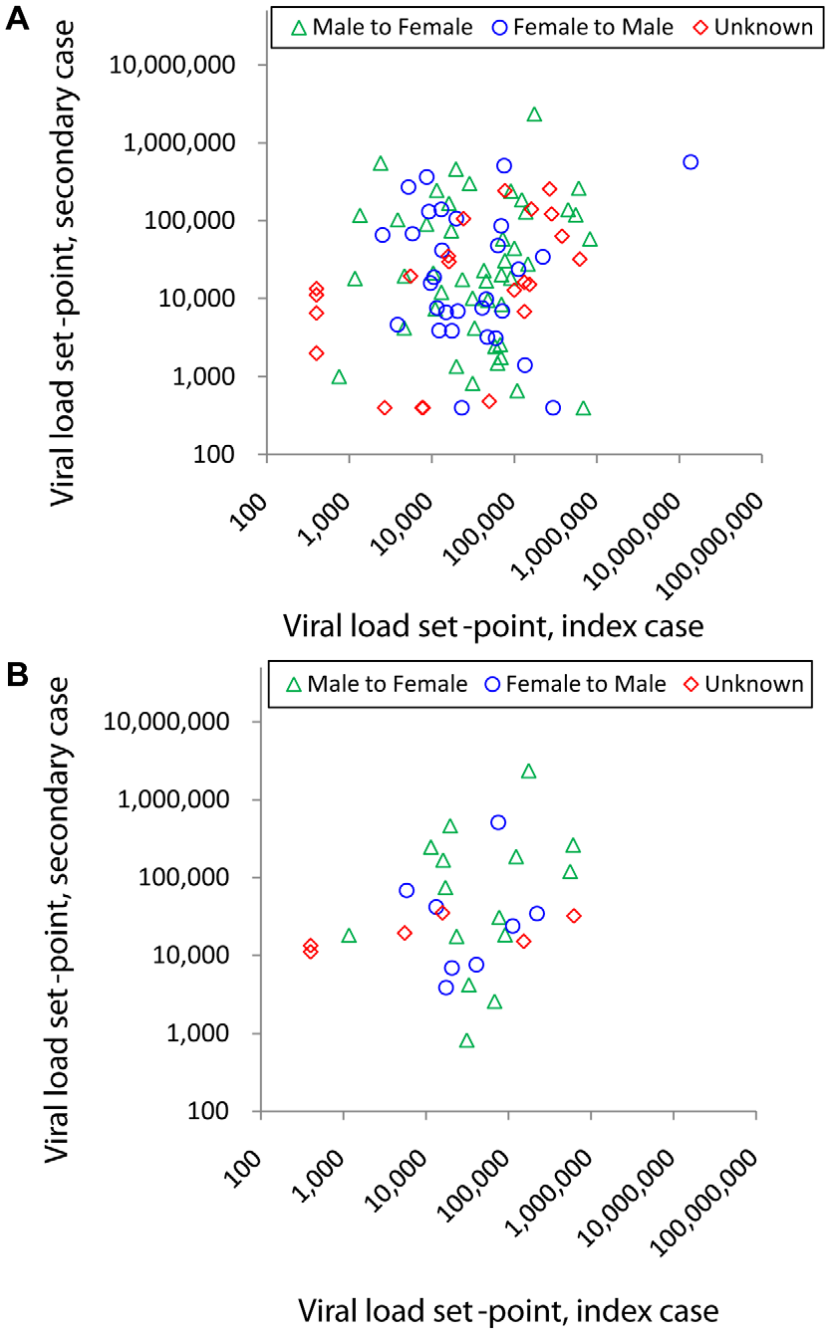
Viral load set-point of index partner versus that of the secondary case in transmitting couples. Couples are stratified by male to female transmission (green triangles), male to female transmission (blue circles) and unknown direction of transmission (red diamonds, plotted as female against male viral load, since the index partner could not be identified). **A** Couples with moderate support for transmission (n = 97). **B** Subgroup of couples with strong genetic support for transmission (n = 29, monophyletic and bootstrap ≥80%). Simple linear regression lines are not shown since this was not the analysis performed.

Viral load set-points of transmitting couples are presented in Figure 2. This data is also presented as the distribution of differences in viral load set-points, Figure S6.

When analysing the 97 couples with moderate support for transmission, the couple effect (which tests whether viral load set-points are similar within couples, see Methods) was found to be borderline significant (*p* = 0.054) by single factor ANOVA. The estimated size of this couple effect was 16%.

In addition, age, GUD and subtype were found to be highly significant (Table 2) in accordance with earlier studies of this cohort [28]. An unexpected finding is that individuals with missing data generally had lower viral loads than other individuals, which suggests there may be some selection bias not captured in this study (Table 1). For this reason, we treated ‘missing data’ as a separate categorical state for the corresponding variables. When the single factor models were instead fitted excluding the missing data, age, GUD and subtype were not found to be significant predictors of viral load.

**Table 2:** Factors which influence viral load set-point.

	Couples with moderate support for transmission (97 couples)			Subgroup of couples with strong support for transmission (29 couples)		
Variable	Coefficient (SE)	*p*	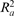	Coefficient (SE)	*p*	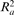
**Single factor models**						
*Couple*		**0.054**	**16%**		**0.067**	**27%**
*Sex*						
Male	0.068 (0.12)	0.58	−0.4%	−0.112 (0.21)	0.92	−1.3%
Female	-			-		
*Age, years*						
40–64	1.11 (0.22)	<0.0001	14%	0.80 (0.67)	0.91	4.7%
30–39	1.08 (0.20)			1.36 (0.57)		
25–29	0.88 (0.20)			1.07 (0.57)		
15–24	0.94 (0.21)			1.04 (0.58)		
Missing	-			-		
*GUD*						
Present	0.65 (0.19)	<0.0001	11%	0.95 (0.40)	0.39	6.58%
Absent	0.70 (0.14)			0.81 (0.36)		
MIssing	-			-		
*Subtype*						
A	1.15 (0.24)	<0.0001	15%	0.37 (0.33)	0.19	3.2%
C	1.33 (0.80)			None		
D	1.16 (0.19)			0.51 (0.26)		
Recombinant	1.01 (0.23)			-		
Missing	-			-		
*Role in transmission for transmitting pairs*						
Index case	0.29 (0.16)	0.17	0.8%	0.46 (0.28)	0.82	2.2%
Secondary case	0.12 (0.16)			0.45 (0.28)		
Unknown	-			-		
**Multiple factor models**						
All factors		**0.0057**	40%		**0.036**	40%
All factors except couple			17%			3%
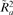			**23%**			**37%**

Individuals were stratified by sex, age, genital ulcer disease (GUD), viral subtype and paired within couples. Regression coefficients, *p*-values and *R^2^* adjusted for degrees of freedom, 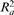, were reported for single factor models. For the model including all factors the *p*-value of the couple effect (see methods) and *R^2^* for the couple effect adjusted for confounders, 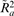, are presented (see Text S2). The analysis was performed for all 97 couples with moderate support for transmission and for a subset of 29 couples with strong support for genetic linkage.

When adjusting for all possible confounders (in a multivariate ANOVA), the couple effect was a significant predictor of viral load set-point (adjusted *p* = 0.0059 for full model). The adjusted estimate for the couple effect was 23% (Table 2). To test the robustness of our conclusions to inclusion of different confounders, we explored all possible combinations of factors (Table S1).

When looking at the subgroup of couples showing strong support for transmission (29 couples), the couple effect was of borderline significance in the unadjusted analysis (*p* = 0.067), but significant when adjusting for confounders (*p* = 0.036). The size of the couple effect was 27%. The couple effect was a key determinant of viral load for most of the multivariate analyses which were performed (Table S2). When adjusting for confounders the estimate of the couple effect increased to 37% (Table 2).

The set of 15 couples for whom there was no support for transmission (i.e. with different serotypes or polyphylectic viral genotypes) might be considered as a small control group for our study. Unfortunately, this group is too small to form a definitive control group. Nonetheless, for completeness, we estimated the couple effect. It was not found to be significant (p = 0.32) and the effect size was smaller (12%).

## Discussion

Our analysis showed that, in this study population, individuals within transmitting couples had similar viral load set-points (*p* = 0.054 in single factor model, *p* = 0.0057 adjusting for confounders) and that this effect explained 16% (23% adjusting for confounders) of the variability in viral load set-points. When the analysis was repeated for the subgroup of couples for whom there was strong genetic support for viral linkage, couples infected with similar viruses also had similar viral load set-points (*p* = 0.067, adjusted *p* = 0.036). The size of the couple effect was estimated to be larger, 27% in single factor model (37% adjusting for confounders), suggesting that the transmitted virus plays a role in determining viral load set-point.

We were unable to assess and account for all possible contributing factors to the correlation of viral loads within couples. Potential confounders include environmental or host factors which could cause couples to have similar viral load set-points. For example, couples may have similar exposure to coinfections or access to health care which might affect viral load set-point.

Besides environmental factors, the viral load set-points of secondary cases could depend on the ‘dose’ of transmitted virus received from the index case. If the dose were to depend on the set-point of the index case, this would lead to correlated viral load set-points. Phylogenetic analysis of 102 early infection isolates indicated that 78 of these infections were established by a single virus, and that the remaining 24 were established by two to five viruses [31]. The viral load set-point of the index partner was not known for that study and therefore the relationship between dose and number of viruses establishing infection is not known. In addition, the relationship between the number of establishing virions and the viral load set-point of the recipient partner is not known. However, since most infections were established by only a few virions, or resulted from the rapid outgrowth of the population descended from these virions, it is likely that the number of infecting virions is similar for a large range of viral load set-points of the infecting partner. Given all these unknown relationships, the hypothesis that a dose effect is driving the observations presented here cannot be discounted, and may be further elucidated by ongoing study in humans and experimental infections of animals [32]–[34].

The most parsimonious explanation for our observation is the existence of viral virulence factors that influence viral load set-point and are partly preserved from one infection to the next. The existence or identity of these viral factors is not well established. Candidate virulence factors include the accumulation of CTL escape mutations at a population level [35], traits determined by viruses preserved on mucosal surfaces by balancing selection [36], and other virulence factors acting by presently unknown mechanisms.

This retrospective study of heterosexual couples in a rural African population suggests that the transmitted virus plays an important role in determining viral load set-point, supporting previous observations [17]. Our study is likely to give an underestimate of the role of viral factors in determining viral load for three main reasons. The infecting viruses in almost all these couples were not identical, only similar, there were only a few viral load measurements per individual and so variability within patients could not be accounted for and we had no information on the host genetics of the infected individuals. Remaining variability in viral load set-point could be due to various host immune factors, coinfections and other environmental factors. The suggestion that the virus plays a role in determining viral load set-point should not negate the importance of host factors [1], [37], but rather implies a complex interaction between host *and* virus.

The similarity of viral load set-points between transmitting couples, as demonstrated in our analysis, have direct implications for potential of HIV-1 virulence to evolve both in untreated infection and in response to public health measures [9]. More extensive studies with greater numbers of couples, more detailed virus and host genetic data and different routes of transmission are required to further test our observation.

## Supporting Information

Text S1 Accession numbers
(0.29 MB PDF)

Text S2 Statistical model and partitioning variance
(0.39 MB PDF)

Figure S1 Clustering of sequences from the couples for whom sequence data was available at both loci, based on concatenated sequences. As Figure 1A, but with all sequences shown.
(0.75 MB PDF)

Figure S2 Clustering of sequences from the couples for whom sequence data was available at *gp41*. As Figure 1B, but with all sequences shown.
(0.76 MB PDF)

Figure S3 Clustering of sequences from the couples for whom sequence data was available at *p24*. As Figure 1C, but with all sequences shown.
(0.76 MB PDF)

Figure S4 Flow diagram for inclusion in the study groups of 97 couples with moderate support for transmission, which includes those with epidemiological linkage together and where available, weak to strong support for transmission (blue); the sub-group of 29 couples with strong support for transmission (green); the 15 couples which genetic evidence suggested did not transmit to each other (red).
(0.58 MB PDF)

Figure S5 Distribution of tree distances. The distribution of tree distances between couples is given for couples for whom there was strong support for transmission (green) and weak genetic support for transmission (blue). In addition, the distribution of tree distances for all other pairwise comparisons between individuals in the trees (Figure 1) is included for comparison (black). For couples with sequences at both loci available the distance shown is that on the concatenated tree. For couples for whom sequence data was only available at one locus, the distance on that single locus tree is used.
(0.01 MB PDF)

Figure S6 Distribution of differences in viral load set-points. The distribution of absolute differences in viral load setpoints for 29 couples with strong support for transmission (green), the remaining 68 couples from the 97 with moderate support for transmission (blue) and all other male to female pairwise comparisons (black).
(0.08 MB PDF)

Table S1 Size of couple effect for different model structures for 97 couples in main analysis. Black circles indicate factors included in the model. The Type III p-value for the couple effect and the adjusted R-squared for the model are given.
(0.13 MB PDF)

Table S2 Size of couple effect for different model structures for subgroup of 29 couples. Black circles indicate factors included in the model. The Type III p-value for the couple effect and the adjusted R-squared for the model are given.
(0.13 MB PDF)
