# Supplementary material for: HIV-1 Transmitting Couples Have Similar Viral Load Set-Points in Rakai, Uganda
Source: PLoS Pathog. 2010 May 6;6(5):e1000876. doi: 10.1371/journal.ppat.1000876 (PMC2865511; doi:10.1371/journal.ppat.1000876)
Supplement: Text S1 — Accession numbers (0.29 MB PDF) [file ppat.1000876.s001.pdf]

HIV-1 transmitting couples have similar viral load set-points in Rakai, Uganda

T. Déirdre Hollingsworth[1], Oliver Laeyendecker[2,3], George Shirreff[1],Christl A. Donnelly[1], David Serwadda[4,6], Maria J. Wawer[5,6], Noah Kiwanuka[4,6], Fred Nalugoda[6] , Aleisha Collinson-Streng[3], Victor Ssempijja[5], William P. Hanage[1], Thomas C. Quinn[2,3], Ronald H. Gray[5], Christophe Fraser[1]

[1] Department of Infectious Disease Epidemiology, Faculty of Medicine, St Mary's Campus, Imperial College London W2 1PG, UK.

[2]Johns Hopkins University, School of Medicine, Baltimore MD 21205, USA.

[3] National Institute of Allergy and Infectious Diseases, NIH, Baltimore, MD, 21205, USA.

[4] School of Public Health, Makerere University, Kampala, Uganda.

[5]Johns Hopkins University, Bloomberg School of Public Health, Baltimore MD, 21205, USA.

[6]Rakai Health Science Program, Uganda Virus Research Institute, Entebbe, Uganda .

Supporting Information: Genbank accession numbers for sequences used.

Couples p24 sequences

GU017802, GQ253766, GU017803, GQ253737, GU017793, GU017799, GU046802, GU017813, GQ252694, GU017806, GU017804, GQ253769, GQ332883, GQ252714, GU017805, GQ252708, GQ253702, AF484494, GQ332965, GU017809, GU017807, GU017815, GU017845, GU017846, GQ252709, GU017795, GU017826, GQ253762, GU017827, GQ333389, GQ333032, GU017829, GU017810, GQ253741, GQ253726, GU017811, GU017808, GQ253727, GU017812, GU017831, GQ253744, GU017791, GU017819, GQ333130, GU017823, GU017841, GU017843, GU017796, GQ253763, GU046803, AF484480, GU017832, GQ252706, GU017814, GQ253677, GU017833, GU017834, GQ253767, GQ333226, AF484497, GU017816, GU017817, GQ333230, GQ252730, GU017842, GQ253788, GU017844, GU017828, GQ253757, GU017818, GQ252738, GQ252722, GU017797, GU017798, GQ253764, GU017837, GQ252715, GU017790, GU017792, GQ252731, GU046805, AF484486, GU017835, GU017800, GQ253730, GU017821, AF484478, GQ253771, GU017830, GQ253774, GU017836, GQ253705, GU046804, GQ252704, GU017822, GU017820, GQ252740, GU017794, GU017801, GQ252721, GU017838, GQ333461, GU017840, GU017825, GU017839, GU017824, GQ253676, GU324974

Couples gp41 sequences

GU046792, GQ253874, GU046793, GQ253857, GU017849, GU017851, GU046794, GU017866, GQ252748, GU017859, GU017855, GQ253869, GU017871, GU017856, GU017894, GU017857, GQ253877, GQ333601, GQ252767, GU017858, GQ252762, GU017879, GQ333669, GU046796, GQ253825, AF484494, GQ333677, GU017862, GU017860, GU017867, GQ333556, GU017854, GU017881, GQ252763, GU017850, GU017882, GQ253871, GU046798, GU017883, GQ334108, GU017895, GU017887, GU017847, GQ333746, GU017885, GQ253846, GU017864, GU017863, GU017861, GQ253847, GU017865, GU046797, GU017876, GQ253801, GU017888, GU017889, GQ253875, GQ333940, GU017868, GU017869, GQ333941, GQ252777, GU017896, GQ253895, GU017899, GU017884, GQ253863, GU046795, GU017897, GQ252779, GU017870,

GQ252787, GU017874, GQ253873, GU017891, GQ252778, GU017877, AF484486, GQ252785, GU017853, GU017848, GQ253850, GU017873, AF484478, GQ253879, GU017852, GU017886, GQ253881, GU017890, GQ253828, GU017898, GQ252758, GU017875, GU017872, GQ252790, GU046799, GU046800, GQ334182, GU017893, GU017880, GU017892, GU017878

Additional p24 sequences,

GQ253666, GQ253667, GQ253668, GQ253669, GQ253670, GQ253671, GQ253672, GQ253674, GQ253678, GQ253679, GQ253680, GQ253681, GQ253682, GQ253683, GQ253684, GQ253685, GQ253686, GQ253687, GQ253691, GQ253693, GQ253694, GQ253695, GQ253697, GQ253698, GQ253699, GQ253700, GQ253701, GQ253703, GQ253704, GQ253706, GQ253707, GQ253709, GQ253710, GQ253713, GQ253714, GQ253716, GQ253717, GQ253718, GQ253720, GQ253721, GQ253722, GQ253724, GQ253728, GQ253729, GQ253731, GQ253732, GQ253733, GQ253734, GQ253735, GQ253736, GQ253752, GQ253754, GQ253755, GQ253756, GQ253758, GQ253759, GQ253760, GQ253768, GQ253770, GQ253773, GQ253775, GQ253776, GQ253777, GQ253778, GQ253779, GQ253780, GQ253781, GQ253782, GQ253783, GQ253784, GQ253785, GQ253786, GQ253787, GQ253789, GQ253790, GQ332768, GQ332769, GQ332770, GQ332771, GQ332774, GQ332776, GQ332777, GQ332778, GQ332783, GQ332785, GQ332786, GQ332787, GQ332788, GQ332789, GQ332790, GQ332794, GQ332796, GQ332797, GQ332799, GQ332800, GQ332801, GQ332802, GQ332805, GQ332807, GQ332809, GQ332810, GQ332811, GQ332813, GQ332815, GQ332816, GQ332817, GQ332818, GQ332819, GQ332820, GQ332823, GQ332829, GQ332830, GQ332833, GQ332834, GQ332835, GQ332836, GQ332840, GQ332841, GQ332842, GQ332843, GQ332844, GQ332848, GQ332849, GQ332850, GQ332851, GQ332853, GQ332854, GQ332856, GQ332858, GQ332859, GQ332860, GQ332861, GQ332862, GQ332864, GQ332865, GQ332866, GQ332867, GQ332869, GQ332870, GQ332871, GQ332872, GQ332873, GQ332877, GQ332878, GQ332879, GQ332881, GQ332882, GQ332884, GQ332889, GQ332890, GQ332892, GQ332893, GQ332894, GQ332895, GQ332896, GQ332897, GQ332898, GQ332900, GQ332901, GQ332902, GQ332904, GQ332905, GQ332908, GQ332909, GQ332910, GQ332911, GQ332912, GQ332913, GQ332914, GQ332915, GQ332916, GQ332917, GQ332918, GQ332919, GQ332922, GQ332923, GQ332924, GQ332925, GQ332927, GQ332928, GQ332929, GQ332932, GQ332936, GQ332937, GQ332938, GQ332939, GQ332941, GQ332943, GQ332946, GQ332947, GQ332951, GQ332954, GQ332955, GQ332956, GQ332957, GQ332958, GQ332959, GQ332962, GQ332963, GQ332968, GQ332969, GQ332970, GQ332971, GQ332974, GQ332978, GQ332979, GQ332980, GQ332981, GQ332982, GQ332983, GQ332985, GQ332986, GQ332987, GQ332988, GQ332992, GQ332995, GQ332996, GQ332997, GQ332998, GQ332999, GQ333000, GQ333001, GQ333004, GQ333005, GQ333007, GQ333008, GQ333009, GQ333010, GQ333013, GQ333015, GQ333016, GQ333017, GQ333018, GQ333019, GQ333021, GQ333022, GQ333024, GQ333025, GQ333027, GQ333028, GQ333031, GQ333033, GQ333034, GQ333035, GQ333038, GQ333039, GQ333040, GQ333042, GQ333043, GQ333044, GQ333046, GQ333047, GQ333048, GQ333050, GQ333053, GQ333056, GQ333058, GQ333059, GQ333060, GQ333061, GQ333063, GQ333067, GQ333068, GQ333071, GQ333072, GQ333074, GQ333076, GQ333080, GQ333081, GQ333086, GQ333088, GQ333089, GQ333090, GQ333091, GQ333094, GQ333095, GQ333096, GQ333097, GQ333100, GQ333101, GQ333104, GQ333105, GQ333106, GQ333107, GQ333108, GQ333111, GQ333116, GQ333125, GQ333126, GQ333129, GQ333131, GQ333133, GQ333137, GQ333138, GQ333139, GQ333140, GQ333141, GQ333142, GQ333144, GQ333147, GQ333148, GQ333152, GQ333153, GQ333156, GQ333160, GQ333162, GQ333164, GQ333165, GQ333166, GQ333168, GQ333169, GQ333170, GQ333171, GQ333172, GQ333173, GQ333174, GQ333175, GQ333177, GQ333179, GQ333180, GQ333181, GQ333183, GQ333185, GQ333186, GQ333187, GQ333190, GQ333191, GQ333192, GQ333193, GQ333194, GQ333197, GQ333198, GQ333199, GQ333201, GQ333202, GQ333207, GQ333208, GQ333210, GQ333211, GQ333212, GQ333213, GQ333214, GQ333215, GQ333216, GQ333218, GQ333219, GQ333220, GQ333221, GQ333222, GQ333223, GQ333225, GQ333227, GQ333229, GQ333231, GQ333232, GQ333235, GQ333236, GQ333238, GQ333239, GQ333241,

GQ333242, GQ333243, GQ333246, GQ333247, GQ333248, GQ333249, GQ333250, GQ333251, GQ333254, GQ333255, GQ333258, GQ333259, GQ333260, GQ333262, GQ333263, GQ333268, GQ333269, GQ333270, GQ333271, GQ333272, GQ333273, GQ333274, GQ333279, GQ333283, GQ333284, GQ333287, GQ333290, GQ333291, GQ333292, GQ333293, GQ333294, GQ333296, GQ333297, GQ333298, GQ333299, GQ333300, GQ333301, GQ333302, GQ333304, GQ333306, GQ333310, GQ333311, GQ333313, GQ333314, GQ333315, GQ333317, GQ333318, GQ333319, GQ333321, GQ333322, GQ333323, GQ333324, GQ333325, GQ333327, GQ333328, GQ333329, GQ333330, GQ333332, GQ333334, GQ333336, GQ333337, GQ333338, GQ333339, GQ333340, GQ333341, GQ333343, GQ333344, GQ333345, GQ333347, GQ333350, GQ333351, GQ333353, GQ333354, GQ333355, GQ333357, GQ333358, GQ333360, GQ333361, GQ333365, GQ333370, GQ333371, GQ333372, GQ333373, GQ333376, GQ333377, GQ333380, GQ333382, GQ333384, GQ333387, GQ333388, GQ333390, GQ333392, GQ333395, GQ333397, GQ333399, GQ333400, GQ333401, GQ333403, GQ333406, GQ333409, GQ333411, GQ333414, GQ333415, GQ333416, GQ333417, GQ333422, GQ333423, GQ333424, GQ333425, GQ333426, GQ333429, GQ333431, GQ333432, GQ333435, GQ333437, GQ333439, GQ333440, GQ333441, GQ333442, GQ333444, GQ333446, GQ333449, GQ333451, GQ333452, GQ333453, GQ333454, GQ333460, GQ333462, GU017748, GU017749, GU017750, GU017751, GU017752, GU017753, GU017754, GU017755, GU017756, GU017757, GU017758, GU017759, GU017760, GU017761, GU017762, GU017763, GU017764, GU017765, GU017766, GU046801, GQ253673, GQ253711, GQ253725

Additional gp41 sequences

GQ333463, GU017767, GQ333465, GQ333466, GQ253791, GQ253792, GQ333474, GQ333475, GQ333477, GQ333478, GQ333484, GQ333485, GQ333486, GQ333487, GQ333488, GQ333489, GQ333490, GQ333494, GQ333500, GQ253793, GQ333502, GQ333504, GQ333506, GQ333508, GQ333509, GQ333510, GQ253794, GQ333511, GQ333514, GQ333516, GQ333517, GQ333520, GQ333522, GQ333523, GQ333525, GQ333526, GU017768, GQ333528, GQ333529, GQ333530, GQ253795, GQ333531, GQ253796, GQ333534, GQ333536, GQ253797, GQ333538, GQ333539, GQ333540, GQ333542, GU017769, GQ333546, GQ333547, GQ333548, GQ333549, GQ333550, GQ333551, GQ253799, GQ333557, GQ333558, GQ333560, GQ333561, GQ333562, GQ333563, GU017770, GQ333564, GQ333565, GQ333566, GQ333568, GQ253802, GQ253803, GQ333569, GQ333572, GQ333574, GQ333575, GQ253804, GQ253805, GQ333576, GQ253806, GQ333582, GQ333583, GQ253807, GQ253808, GQ333588, GQ333589, GQ333590, GU017771, GQ333595, GQ253809, GQ253810, GQ333596, GQ333598, GQ333600, GQ333602, GQ253811, GQ333605, GQ333606, GQ333609, GQ333610, GQ333611, GQ333612, GQ333614, GQ333615, GQ333616, GQ333618, GQ333620, GQ333621, GQ333622, GQ333623, GQ253814, GQ333624, GQ333625, GQ333626, GQ333627, GQ333628, GQ333629, GQ333630, GQ333631, GQ333632, GQ333633, GQ333634, GQ333635, GQ333638, GQ253816, GQ333639, GQ333640, GQ333641, GQ333642, GU017772, GQ253817, GQ333643, GQ253818, GQ333645, GQ253820, GQ333647, GQ253821, GU017773, GQ333652, GQ333653, GQ253822, GQ333654, GQ333656, GQ333657, GQ333658, GQ253823, GQ253824, GQ333661, GU017774, GQ333665, GQ333666, GQ333667, GQ333668, GU017775, GQ333670, GQ333673, GQ333675, GQ253826, GQ333678, GQ333679, GQ253827, GQ333680, GQ333681, GQ333682, GQ333683, GQ333685, GQ253829, GQ333688, GQ333690, GQ333691, GQ333692, GU017776, GQ253830, GQ333693, GQ253831, GQ333694, GQ333695, GQ333696, GQ333697, GQ333698, GQ333700, GQ333704, GQ253833, GQ253834, GQ333708, GQ333709, GQ333710, GQ333711, GQ333713, GQ333715, GQ333718, GQ333719, GQ333720, GQ333721, GQ333722, GQ333723, GQ333725, GQ333727, GQ333728, GQ253836, GQ333729, GQ333730, GQ333732, GQ253837, GQ333733, GQ333735, GQ253838, GQ333737, GQ253840, GQ333738, GQ333740, GQ333741, GQ253841, GQ333745, GQ333747, GQ333748, GQ333751, GQ333754, GQ333755, GQ333756, GQ333758, GQ253842, GQ333760, GQ333761, GQ333764, GQ333765, GQ333767, GQ333769, GQ333771, GQ333773, GQ253844, GQ333775, GQ333776, GQ333777, GQ333778, GQ333780, GQ333783, GQ253848, GQ253849, GQ333784, GQ333785, GQ253851, GQ333789, GQ333790, GQ333791, GQ333793, GQ333794, GQ333795, GQ253852,

GQ253853, GQ333800, GQ333804, GQ333805, GQ333806, GQ333808, GQ333812, GQ333813, GQ333815, GQ333816, GQ253854, GQ333818, GQ333819, GQ333821, GQ333822, GQ253855, GQ333823, GQ333824, GQ333825, GQ333826, GQ333830, GQ253856, GQ333834, GQ333835, GQ333837, GU017777, GQ333841, GQ333843, GQ333844, GQ333845, GQ333846, GQ333847, GQ333849, GQ333851, GQ333852, GQ333855, GQ333857, GU017778, GQ333860, GQ333861, GU017779, GQ333863, GQ333864, GQ333866, GQ333867, GQ333869, GQ333871, GQ333873, GQ333874, GQ333875, GQ333876, GQ333878, GQ333879, GQ333880, GQ333882, GQ333884, GQ333885, GQ333887, GQ333888, GQ333889, GQ333891, GQ333893, GQ333894, GQ253858, GU017780, GQ333896, GQ333897, GQ333898, GQ333900, GQ333901, GQ333902, GQ333903, GQ333904, GQ333905, GQ333906, GQ253860, GQ333912, GQ333913, GQ333919, GQ333920, GQ333921, GQ333922, GQ333923, GQ253861, GQ333925, GQ333926, GQ333927, GQ333929, GQ333930, GQ333932, GQ333934, GQ333936, GQ333938, GQ333939, GU017781, GQ333942, GQ333943, GQ253862, GQ333947, GQ333949, GQ333951, GQ333952, GQ333953, GQ333954, GU017782, GQ333957, GQ333958, GQ333959, GQ333961, GQ333963, GQ333964, GQ333966, GQ333967, GQ333971, GQ333973, GQ333974, GQ333977, GQ333979, GQ253864, GQ333981, GQ333982, GQ333983, GQ333984, GQ333985, GU017783, GQ333988, GQ333989, GQ253865, GU017784, GQ253866, GQ333996, GQ333997, GQ333998, GQ334000, GQ334003, GQ334005, GQ334006, GQ334007, GQ334008, GQ253870, GQ334010, GQ334013, GQ334014, GQ334015, GQ334016, GQ334017, GQ334020, GQ334021, GQ334023, GQ334026, GQ334027, GQ334031, GQ334032, GU017785, GU017786, GQ334034, GQ334035, GU017787, GQ334036, GQ334037, GQ334038, GQ334040, GQ334041, GQ334042, GQ334043, GU017788, GQ334046, GQ334049, GQ334051, GQ334052, GQ334053, GQ334054, GQ334055, GQ334057, GQ334058, GQ334059, GQ253876, GQ334061, GQ334062, GQ334066, GQ334067, GQ334068, GQ334070, GQ334071, GQ334072, GQ334073, GQ334074, GQ334075, GQ334078, GQ334079, GQ334082, GQ334084, GQ334086, GQ334087, GQ253878, GQ334089, GQ334091, GU017789, GQ334094, GQ334095, GQ334097, GQ334101, GQ334102, GQ253880, GQ334105, GQ334106, GQ253882, GQ253883, GQ334109, GQ334112, GQ334113, GQ334115, GQ334117, GQ334118, GQ334119, GQ253884, GQ334121, GQ253885, GQ334123, GQ253886, GQ334124, GQ253887, GQ334126, GQ334129, GQ334130, GQ253888, GQ334131, GQ334132, GQ334139, GQ334140, GQ334141, GQ334142, GQ334143, GQ334148, GQ253889, GQ334151, GQ334152, GQ253890, GQ334157, GQ334158, GQ253891, GQ334159, GQ334160, GQ334161, GQ334162, GQ334163, GQ334166, GQ334167, GQ253892, GQ253893, GQ334171, GQ253894, GQ334173, GQ253896, GQ253897, GQ334174, GQ334175, GQ334176, GQ334181, GQ334183, GU017769, GQ253832, GQ253845
