# Supplementary material for: HIV-1 Transmitting Couples Have Similar Viral Load Set-Points in Rakai, Uganda
Source: PLoS Pathog. 2010 May 6;6(5):e1000876. doi: 10.1371/journal.ppat.1000876 (PMC2865511; doi:10.1371/journal.ppat.1000876)
Supplement: Text S2 — Statistical model and partitioning variance (0.39 MB PDF) [file ppat.1000876.s002.pdf]

HIV-1 transmitting couples have similar viral load set-points in Rakai, Uganda

T. Déirdre Hollingsworth[1], Oliver Laeyendecker[2,3], George Shirreff[1],Christl A. Donnelly[1], David Serwadda[4,6], Maria J. Wawer[5,6], Noah Kiwanuka[4,6], Fred Nalugoda[6] , Aleisha Collinson-Streng[3], Victor Ssempijja[5], William P. Hanage[1], Thomas C. Quinn[2,3], Ronald H. Gray[5], Christophe Fraser[1]

[1] *Department of Infectious Disease Epidemiology, Faculty of Medicine, St Mary's Campus, Imperial College London W2 1PG, UK.*

[2]*Johns Hopkins University, School of Medicine, Baltimore MD 21205, USA.*

[3] *National Institute of Allergy and Infectious Diseases, NIH, Baltimore, MD, 21205, USA.*

[4] *School of Public Health, Makerere University, Kampala, Uganda.*

[5]*Johns Hopkins University, Bloomberg School of Public Health, Baltimore MD, 21205, USA.*

[6]*Rakai Health Science Program, Uganda Virus Research Institute, Entebbe, Uganda .*

Supporting Text

1. Model description

The multiple factor model including all factors reported in Table 2 results in a unique prediction for each individual based on which couple they are in and their personal characteristics. Such that, for example, for person *i* in couple *j* of the 97 couples, the expected value of their log10 viral load (L_{ij}) is given by the following equation:

$$L_{ij} = C_j - 0.15M_{ij}$$

$$-0.15A_{15\text{-}24ij} - 0.32\,A_{25\text{-}29ij} + 0.35\,A_{30\text{-}39ij} + 0.73\,A_{40\text{-}64ij}$$

$$0.42G_{pij} + 0.62\,G_{nij}$$

$$0.068A_{ij} + 1.85C_{ij} + 0.39D_{ij} + 0.78R_{ij}$$

$$+0.42I_{1ij} + 0.00I_{2ij}$$

where C_j is the intercept associated with couple j; M_{ij}=1 if individual i in couple j is male and 0 otherwise; $A_{15\text{-}24ij}$=1 if individual i in couple j is aged between 15 and 24 and 0 otherwise ($A_{25\text{-}29ij}$, $A_{30\text{-}39ij}$ and $A_{40\text{-}64ij}$ are similarly defined for those aged 25-29, 30-39 and 40-64 respectively). G_{pij}=1 if individual i in couple j is coinfected with a GUD and 0 otherwise, G_{nij}=1 if individual i in couple j is not coinfected with a GUD and 0 otherwise; A_{ij}=1 if individual i in couple j is infected with a subtype A virus and 0 otherwise, D_{ij}, C_{ij} and R_{ij} are similarly defined for subtypes D and C and recombinant viruses respectively; I_{1ij}=1 if individual i in couple j is the index partner and 0 otherwise, I_{2ij} if they are the secondary transmission case and 0 otherwise. Values of C_j ranged from 2.60 to 7.13.

2. Estimate of heritability adjusted for confounders

The adjusted R^2, notated R_a^2, in the single factor analysis for the couple effect adjusts the R^2 for the number of degrees of freedom in any analysis. In addition to adjusting for degrees of freedom, the estimate of heritability should be adjusted for the effect of the other variables (gender, GUD status, age, subtype and role in transmission). This adjusted estimate for the contribution of the couple effect to variation in viral load set point is estimated by partitioning the variance by the method described below, described in detail by Legendre and Legendre (1998).

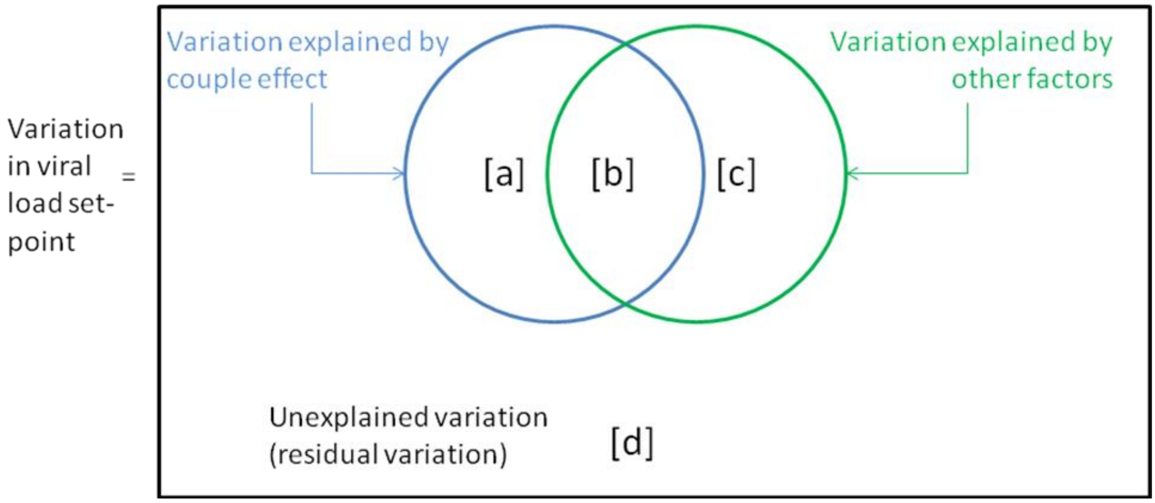

Figure ST1 Partitioning of the variance of the response variable, viral load set-point, among two sets of explanatory variables, couple effect and other factors (gender, age etc). The rectangle represents the variation in viral load set-point accounted for by the linear models and residual. Fraction [b] is the intersection (*not* the interaction) of the amounts of variation explained by linear models of these two groups of effects. (adapted from Figure 10.10 of Legendre & Legendre,1998).

The variation in viral load set-point (Figure ST1 rectangle) can be partitioned into that which is explained by the linear models which take account of known variables (within circles), and that which is not (residual variance, outside the circles). Of the variance which is explained by a variable, or group of variables, some of this may also be explained by other variables in the model (Figure ST1 area [b]). The portion of variance which is explained by each factor alone must therefore be calculated from the model accounting for all variables minus that which is accounted by all variables except the variable of interest (Figure ST1 [a] = [a+b+c] – [b+c]). This adjustment to the adjusted R-square to account for confounding variables is called the adjusted semi-partial R-square, which we notate $\tilde{R}_a^2$. The calculations for our dataset are shown in Table ST1 below.

Table ST1 Partitioning the variance in viral load set-point due to couple effect and other factors for the linear models fitted to 97 couples with moderate or strong support for transmission and for the subset of 29 couples with strong support for transmission. Adapted from Legendre and Legendre (1998).

		Couples with moderate support for transmission (97 couples)				Subgroup of couples with strong support for transmission (29 couples)			
**Description**	**Fractions of variation**	R^2	*data*	*parameters*	R_a^2	R^2	*data*	*parameters*	R_a^2
Model accounting for all factors	[a+b+c]	*74%*	*194*	*108*	40%	*77%*	*194*	*35*	40%
Model accounting for all factors except couple effect	[b+c]	*23%*	*194*	*13*	17%	*22%*	*194*	*11*	3%
Couple effect single factor model	[a+b]	*58%*	*194*	*96*	16%	*63%*	*194*	*28*	27%
**Partitioned variance**					$\tilde{R}_a^2$				$\tilde{R}_a^2$
Couple effect	[a]			40%-17% =	23%			40%-3% =	37%
Other factors	[c]			40%-16% =	24%			40%-27% =	13%
Intersection	[b]			40%-23%-24% =	-6%*			40%-37%-13% =	-9%*
Residuals	[d]			100%-23%-24%-(-6%) =	60%			100%-37%-13%-(-9%) =	60%
Total	[a+b+c+d]				100%				100%

*Note that a negative value for the intersection, [b], indicates that two groups of variables, together, explain the variation in viral load set-point better than the sum of the separate effects of these groups of variables.

Reference

Legendre, P. and L. Legendre (1998). Numerical Ecology, Elsevier, Amsterdam, The Netherlands.
