# Supplementary figures and images for: HIV-1 Transmitting Couples Have Similar Viral Load Set-Points in Rakai, Uganda
Source: PLoS Pathog. 2010 May 6;6(5):e1000876. doi: 10.1371/journal.ppat.1000876 (PMC2865511; doi:10.1371/journal.ppat.1000876)

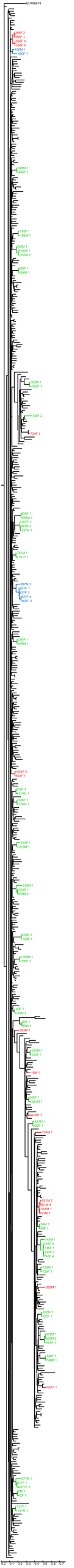

Supplement: Figure S1 — Clustering of sequences from the couples for whom sequence data was available at both loci, based on concatenated sequences. As Figure 1A, but with all sequences shown. (0.75 MB PDF) [file ppat.1000876.s003.pdf]

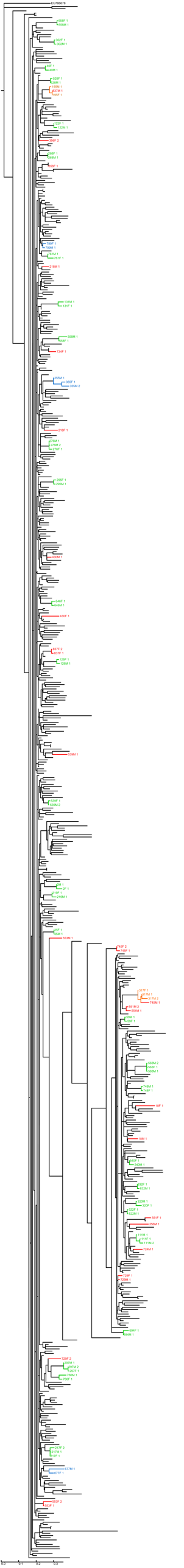

Supplement: Figure S2 — Clustering of sequences from the couples for whom sequence data was available at gp41. As Figure 1B, but with all sequences shown. (0.76 MB PDF) [file ppat.1000876.s004.pdf]

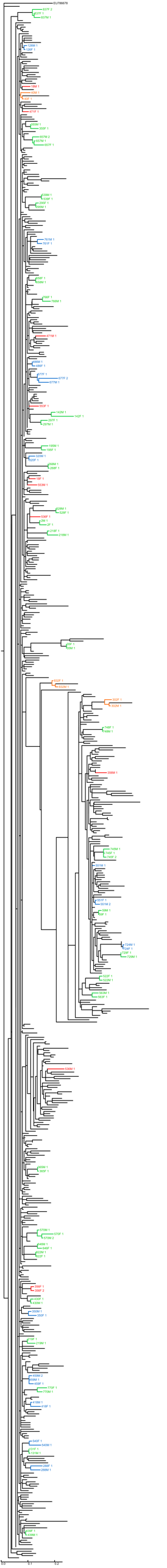

Supplement: Figure S3 — Clustering of sequences from the couples for whom sequence data was available at p24. As Figure 1C, but with all sequences shown. (0.76 MB PDF) [file ppat.1000876.s005.pdf]

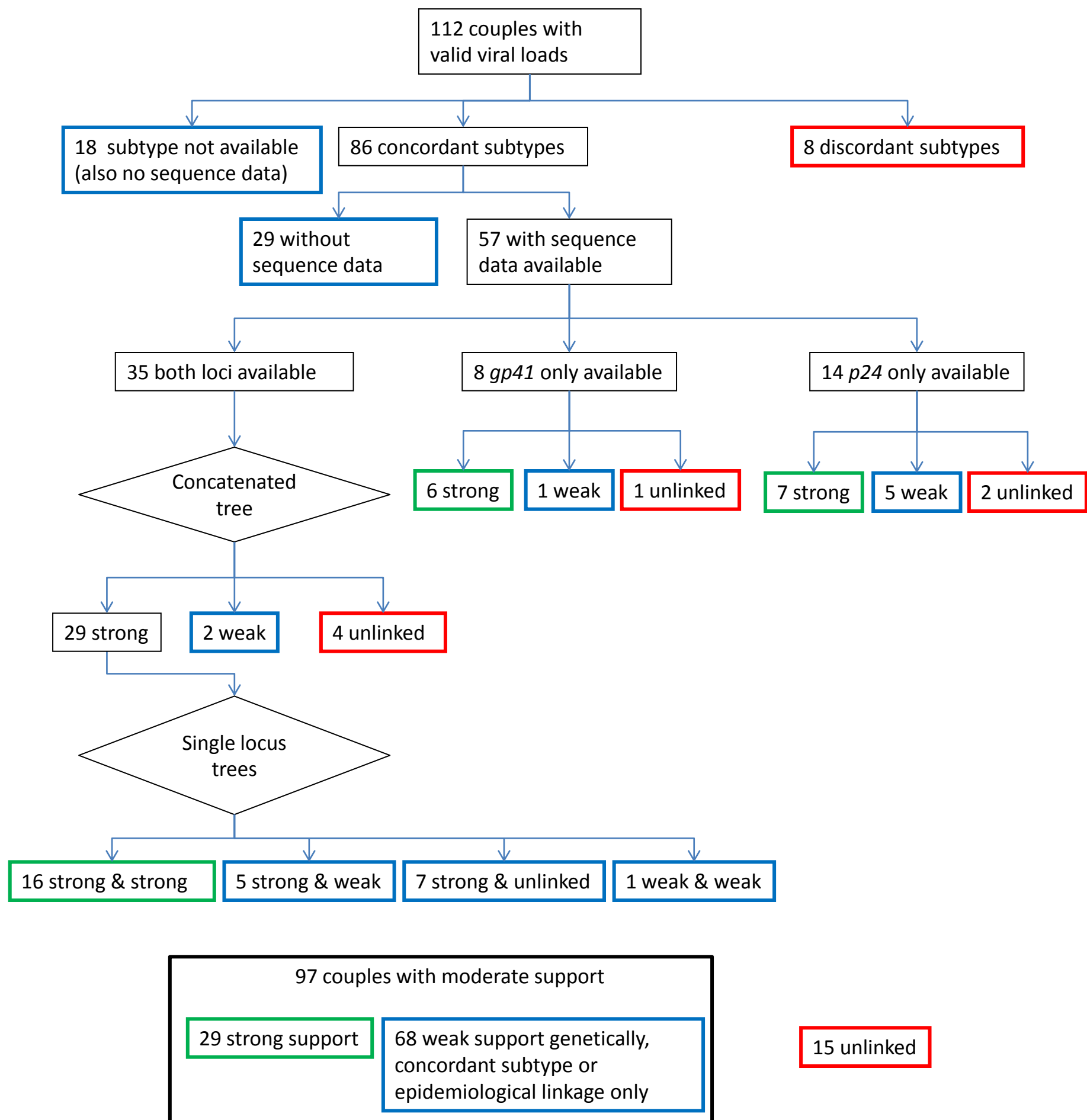

Supplement: Figure S4 — Flow diagram for inclusion in the study groups of 97 couples with moderate support for transmission, which includes those with epidemiological linkage together and where available, weak to strong support for transmission (blue); the sub-group of 29 couples with strong support for transmission (green); the 15 couples which genetic evidence suggested did not transmit to each other (red). (0.58 MB PDF) [file ppat.1000876.s006.pdf]

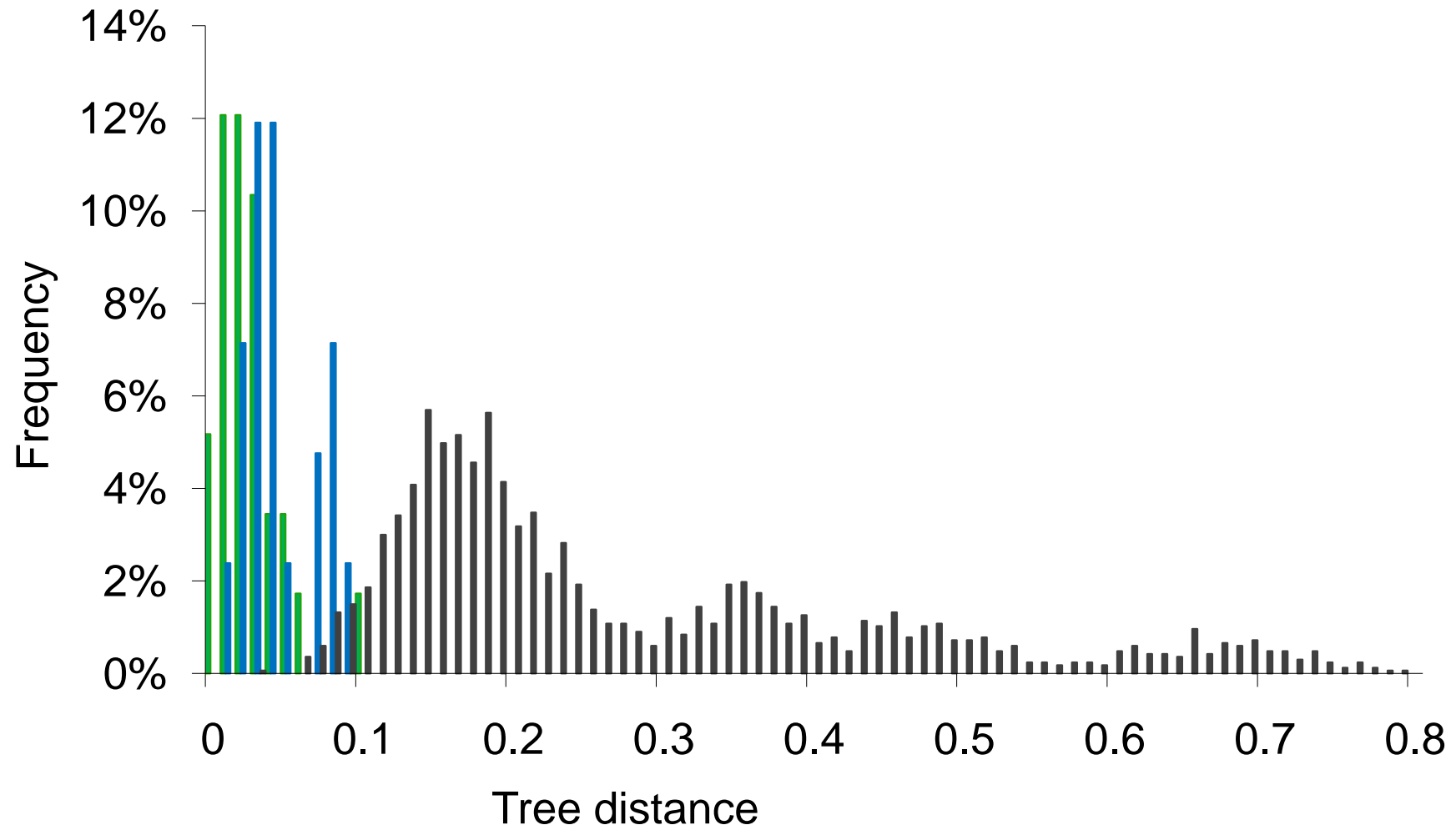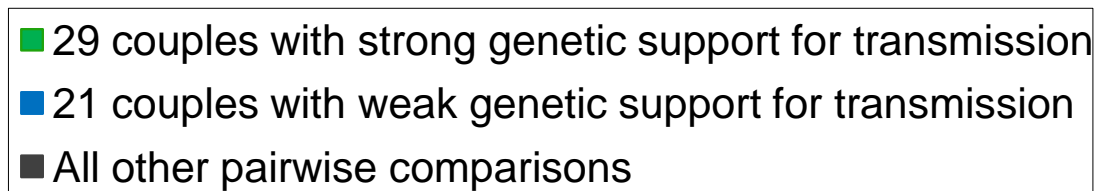

Supplement: Figure S5 — Distribution of tree distances. The distribution of tree distances between couples is given for couples for whom there was strong support for transmission (green) and weak genetic support for transmission (blue). In addition, the distribution of tree distances for all other pairwise comparisons between individuals in the trees (Figure 1) is included for comparison (black). For couples with sequences at both loci available the distance shown is that on the concatenated tree. For couples for whom sequence data was only available at one locus, the distance on that single locus tree is used. (0.01 MB PDF) [file ppat.1000876.s007.pdf]
