# Supplementary material for: HIV-1 Transmitting Couples Have Similar Viral Load Set-Points in Rakai, Uganda
Source: PLoS Pathog. 2010 May 6;6(5):e1000876. doi: 10.1371/journal.ppat.1000876 (PMC2865511; doi:10.1371/journal.ppat.1000876)
Supplement: Figure S6 — Distribution of differences in viral load set-points. The distribution of absolute differences in viral load setpoints for 29 couples with strong support for transmission (green), the remaining 68 couples from the 97 with moderate support for transmission (blue) and all other male to female pairwise comparisons (black). (0.08 MB PDF) [file ppat.1000876.s008.pdf]

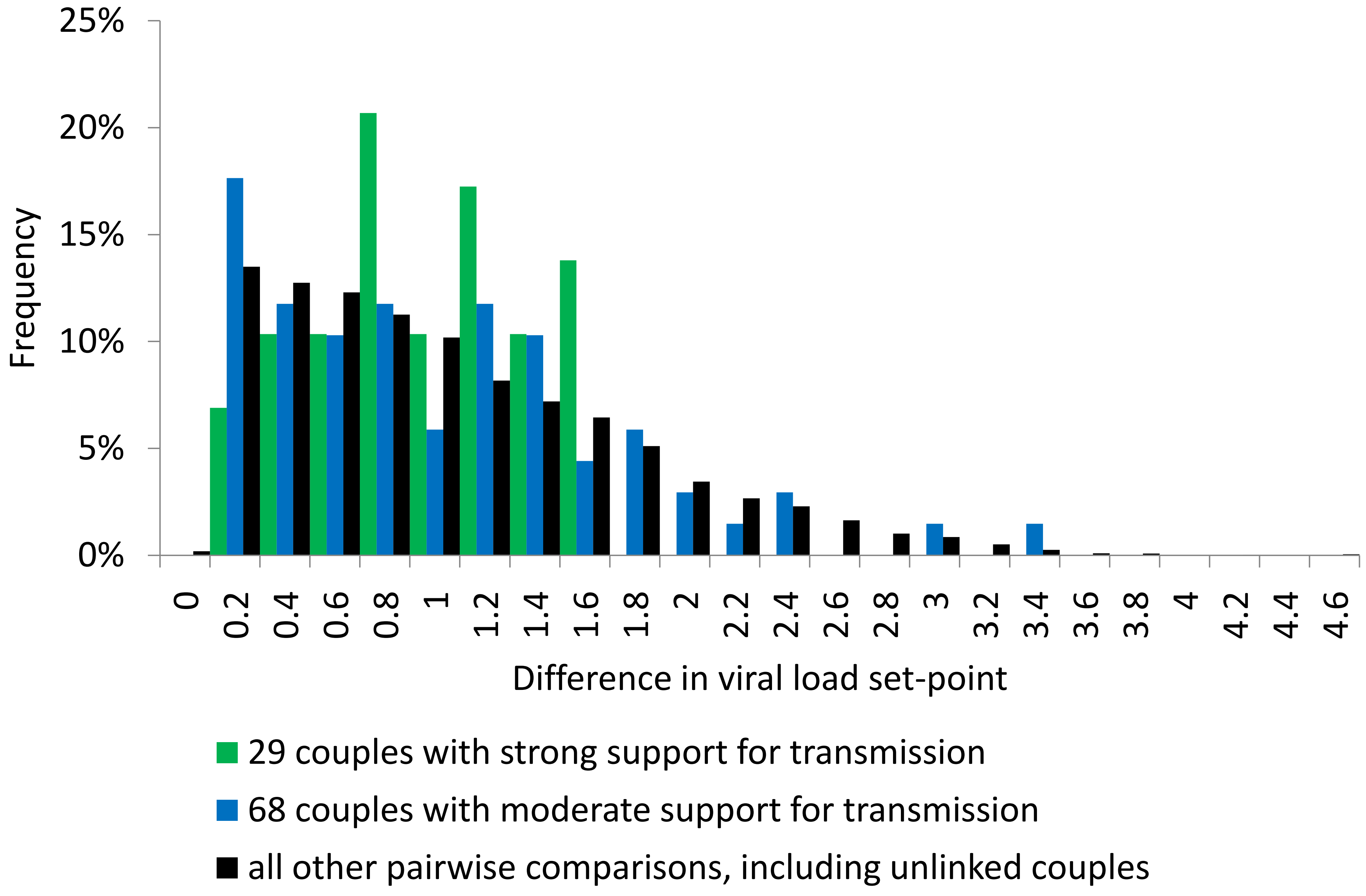

25%
20%
15%
10%
5%
0%
Frequency
0
0.2
0.4
0.6
0.8
1
1.2
1.4
1.6
1.8
2
2.2
2.4
2.6
2.8
3
3.2
3.4
3.6
3.8
4
4.2
4.4
4.6
Difference in viral load set-point
29 couples with strong support for transmission
68 couples with moderate support for transmission
all other pairwise comparisons, including unlinked couples
