# Supplementary material for: HIV-1 Transmitting Couples Have Similar Viral Load Set-Points in Rakai, Uganda
Source: PLoS Pathog. 2010 May 6;6(5):e1000876. doi: 10.1371/journal.ppat.1000876 (PMC2865511; doi:10.1371/journal.ppat.1000876)
Supplement: Table S1 — Size of couple effect for different model structures for 97 couples in main analysis. Black circles indicate factors included in the model. The Type III p-value for the couple effect and the adjusted R-squared for the model are given. (0.13 MB PDF) [file ppat.1000876.s009.pdf]

Table S1. Size of couple effect for different model structures for 97 couples in main analysis. Black circles indicate factors included in the model. The Type III p-value for the couple effect and the adjusted R-squared for the model are given.

Factors included in the model							
Couple	Gender	Age	Subtype	GUD	Role in transmission	p-value for couple effect	Adjusted R-squared
●	❍	●	❍	●	●	0.0039	40%
●	●	●	❍	●	●	0.0042	40%
●	●	●	●	●	●	0.0059	40%
●	❍	●	●	●	●	0.0068	40%
●	●	●	●	❍	●	0.0082	36%
●	❍	●	❍	❍	●	0.0070	36%
●	●	●	❍	❍	●	0.0069	36%
●	❍	●	●	❍	●	0.0105	35%
●	❍	❍	●	●	●	0.0212	35%
●	●	❍	●	●	●	0.0244	34%
●	❍	❍	❍	●	●	0.0134	34%
●	❍	●	❍	●	❍	0.0171	34%
●	●	❍	❍	●	●	0.0148	34%
●	❍	●	❍	❍	❍	0.0122	34%
●	❍	●	●	●	❍	0.0260	34%
●	❍	●	●	❍	❍	0.0178	33%
●	●	●	❍	●	❍	0.0202	33%
●	●	●	●	❍	❍	0.0180	33%
●	●	●	❍	❍	❍	0.0139	33%
●	●	●	●	●	❍	0.0283	33%
●	❍	❍	●	●	❍	0.0471	31%
●	❍	❍	●	❍	●	0.0343	31%
●	●	❍	●	●	❍	0.0529	30%
●	❍	❍	●	❍	❍	0.0411	30%
●	●	❍	●	❍	●	0.0384	30%
●	●	❍	●	❍	❍	0.0464	29%
●	●	❍	❍	●	❍	0.0456	27%
●	❍	❍	❍	●	❍	0.0453	27%
❍	❍	●	●	●	●		18%
❍	❍	❍	●	●	●		17%
❍	❍	●	❍	●	●		17%
❍	●	●	●	●	●		17%
●	❍	❍	❍	❍	●	0.0530	17%
❍	●	❍	●	●	●		17%
❍	●	●	❍	●	●		17%
●	●	❍	❍	❍	●	0.0585	16%
●	❍	❍	❍	❍	❍	0.0541	16%
❍	❍	❍	●	●	❍		16%
❍	❍	●	●	●	❍		16%
❍	●	❍	●	●	❍		16%
●	●	❍	❍	❍	❍	0.0583	16%
❍	●	●	●	●	❍		15%
❍	❍	●	❍	●	❍		15%
❍	❍	❍	❍	●	●		15%
❍	●	●	❍	●	❍		15%
❍	❍	❍	●	❍	❍		15%
❍	●	❍	❍	●	●		15%
❍	❍	❍	●	❍	●		15%
❍	●	❍	●	❍	❍		14%
❍	❍	●	●	❍	❍		14%
❍	❍	●	●	❍	●		14%
❍	●	❍	●	❍	●		14%
❍	●	●	●	❍	❍		14%
❍	❍	●	❍	❍	●		14%

Factors included in the model							
Couple	Gender	Age	Subtype	GUD	Role in transmission	p-value for couple effect	Adjusted R-squared
❍	❍	●	❍	❍	❍		14%
❍	●	●	●	❍	●		14%
❍	●	●	❍	❍	❍		13%
❍	●	●	❍	❍	●		13%
❍	❍	❍	❍	●	❍		11%
❍	●	❍	❍	●	❍		11%
❍	❍	❍	❍	❍	❍		1%
❍	●	❍	❍	❍	●		0%
❍	●	❍	❍	❍	❍		0%
