# Supplementary material for: HIV-1 Transmitting Couples Have Similar Viral Load Set-Points in Rakai, Uganda
Source: PLoS Pathog. 2010 May 6;6(5):e1000876. doi: 10.1371/journal.ppat.1000876 (PMC2865511; doi:10.1371/journal.ppat.1000876)
Supplement: Table S2 — Size of couple effect for different model structures for subgroup of 29 couples. Black circles indicate factors included in the model. The Type III p-value for the couple effect and the adjusted R-squared for the model are given. (0.13 MB PDF) [file ppat.1000876.s010.pdf]

Table S2. Size of couple effect for different model structures for the 29 couples. Black circles indicate factors included in the model. The Type III p-value for the couple effect and the adjusted R-squared for the model are given.

Factors included in the model							
Couple	Gender	Age	Subtype	GUD	Role in transmission	p-value for couple effect	Adjusted R-squared
●	●	●	●	●	●	0.0359	40% *
●	●	●	❍	●	●	0.0436	40% *
●	●	●	●	❍	●	0.0285	39% *
●	●	●	❍	❍	●	0.0366	39% *
●	●	●	●	❍	❍	0.0365	36% *
●	●	●	❍	❍	❍	0.0447	36% *
●	❍	●	●	●	●	0.0560	36% *
●	❍	●	❍	●	●	0.0662	36% *
●	❍	●	●	❍	❍	0.0370	36% *
●	❍	●	❍	❍	❍	0.0453	36% *
●	❍	●	●	❍	●	0.0447	35% *
●	❍	●	❍	❍	●	0.0564	35% *
●	❍	●	●	●	❍	0.0607	33% *
●	❍	●	❍	●	❍	0.0742	33% *
●	●	●	●	●	❍	0.0643	32% *
●	●	●	❍	●	❍	0.0781	32% *
●	❍	❍	●	●	❍	0.1101	28% *
●	❍	❍	❍	●	❍	0.1189	28%
●	❍	❍	●	❍	❍	0.0832	27% *
●	❍	❍	❍	❍	❍	0.0672	27%
●	❍	❍	●	●	●	0.1559	26% *
●	❍	❍	❍	●	●	0.1767	26% *
●	●	❍	●	❍	❍	0.0943	26% *
●	●	❍	❍	❍	❍	0.0771	26%
●	●	❍	●	●	❍	0.1322	25% *
●	●	❍	❍	●	❍	0.1425	25%
●	❍	❍	●	❍	●	0.1081	25% *
●	❍	❍	❍	❍	●	0.1086	25% *
●	●	❍	●	●	●	0.1833	23% *
●	●	❍	❍	●	●	0.2066	23% *
●	●	❍	●	❍	●	0.1210	23% *
●	●	❍	❍	❍	●	0.1218	23% *
❍	❍	❍	❍	●	●		10%
❍	❍	❍	●	●	●		8%
❍	●	❍	❍	●	●		8%
❍	❍	❍	❍	●	❍		7%
❍	❍	●	❍	●	●		7%
❍	●	❍	●	●	●		6%
❍	❍	❍	●	●	❍		6%
❍	❍	●	❍	❍	●		5%
❍	●	●	❍	●	●		5%
❍	❍	●	●	●	●		5%
❍	●	●	❍	❍	●		5%
❍	●	❍	❍	●	❍		5%
❍	❍	●	❍	❍	❍		5%
❍	❍	●	❍	●	❍		4%
❍	●	❍	●	●	❍		4%
❍	●	●	❍	❍	❍		4%
❍	●	●	●	●	●		3%
❍	❍	❍	●	❍	❍		3%
❍	❍	●	●	❍	●		3%
❍	❍	●	●	❍	❍		3%
❍	●	●	❍	●	❍		3%
❍	❍	❍	●	❍	●		3%

Factors included in the model							
Couple	Gender	Age	Subtype	GUD	Role in transmission	p-value for couple effect	Adjusted R-squared
❍	●	●	●	❍	●		2%
❍	❍	❍	❍	❍	❍		2%
❍	❍	●	●	●	❍		2%
❍	●	●	●	❍	❍		2%
❍	●	❍	●	❍	❍		2%
❍	●	❍	●	❍	●		1%
❍	●	❍	❍	❍	●		1%
❍	●	●	●	●	❍		0%
❍	●	❍	❍	❍	❍		-1%

* in fitting this model to the data, some parameters were set to zero, reducing the number of parameters for calculation of adjusted R-squared.
